# Association of serum vitamin D and parathyroid hormone with subclinical atherosclerotic phenotypes: The Dong-gu Study

**DOI:** 10.1371/journal.pone.0186421

**Published:** 2017-10-31

**Authors:** Young-Hoon Lee, Sun-Seog Kweon, Jin-Su Choi, Hae-Sung Nam, Kyeong-Soo Park, Seong-Woo Choi, So-Yeon Ryu, Su-Hyun Oh, Min-Ho Shin

**Affiliations:** 1 Department of Preventive Medicine & Institute of Wonkwang Medical Science, Wonkwang University School of Medicine, Iksan, Jeonbuk, Republic of Korea; 2 Regional Cardiocerebrovascular Center, Wonkwang University Hospital, Iksan, Jeonbuk, Republic of Korea; 3 Department of Preventive Medicine, Chonnam National University Medical School, Hwasun, Republic of Korea; 4 Jeonnam Regional Cancer Center, Chonnam National University Hwasun Hospital, Hwasun, Republic of Korea; 5 Department of Preventive Medicine, Chungnam National University School of Medicine, Daejeon, Republic of Korea; 6 Department of Preventive Medicine, Seonam University College of Medicine, Namwon, Jeonbuk, Republic of Korea; 7 Department of Preventive Medicine, Chosun University Medical School, Gwangju, Republic of Korea; University of Alabama at Birmingham, UNITED STATES

## Abstract

**Background:**

Although previous studies reported an association between serum vitamin D and parathyroid hormone (PTH) with carotid atherosclerosis or arterial stiffness, these were inconsistent. We examined the independent association between serum vitamin D and PTH with multiple subclinical markers of atherosclerosis.

**Methods:**

A total of 8,217 subjects who participated in the Dong-gu Study in Korea were included in the final analysis. The carotid artery structure, including intima-media thickness (IMT), plaques, and luminal diameter, was evaluated using a high-resolution B-mode ultrasound. The brachial-ankle pulse wave velocity (baPWV) was determined using an automatic waveform analysis device, and the mean of the left and right baPWV was used.

**Results:**

The PTH concentration was positively associated with carotid luminal diameter and baPWV, but not with carotid IMT and plaques. The mean carotid luminal diameter of individuals with PTH levels in the second, third, and fourth quartiles was significantly larger compared with those in the first quartile (*P*-trend < 0.01). The mean baPWV of individuals with PTH levels in the fourth quartile was significantly greater than those with PTH levels in the first quartile (*P*-trend = 0.01). However, there was no significant association between vitamin D and any atherosclerotic phenotypes, including carotid IMT, plaques, luminal diameter, and baPWV.

**Conclusion:**

This suggests that PTH might affect the development of atherosclerosis by altering vascular compliance.

## Introduction

Numerous epidemiological studies have demonstrated associations between lower serum vitamin D and higher parathyroid hormone (PTH) concentrations with cardiovascular events and deaths [1–6]. Although several explanations for the relationships between serum vitamin D, PTH, and cardiovascular disease have been presented, the underlying mechanisms remain unclear. One possible explanation is that atherosclerosis might mediate a link between serum vitamin D, PTH and cardiovascular disease (CVD).

Carotid atherosclerotic indices, including intima-media thickness (IMT) and plaques, are significant risk factors for predicting CVD [7–9]. Carotid enlargement is measured using the luminal diameter and is a useful indicator of carotid atherosclerosis [10]. It has been considered to be a surrogate end point of cardiovascular events [11–13]. Conversely, brachial-ankle pulse wave velocity (baPWV), a surrogate marker of arterial stiffness that reflects arterial compliance, is an independent predictor of CVD [14,15].

To date, although some epidemiological studies have reported an association between serum vitamin D or PTH with carotid atherosclerosis or arterial stiffness, the associations are inconsistent. Several studies demonstrated that serum vitamin D levels were inversely associated with carotid IMT, plaque thickness, coronary artery stenosis, or pulse wave velocity [16–19], whereas others reported no association between serum vitamin D and carotid IMT or plaques [20–22]. In addition, some studies revealed that serum PTH was positively associated with carotid IMT [23], whereas other found no associations between serum PTH and carotid IMT or plaques [16,20].

Only a small number of studies have examined the independent association between both serum vitamin D and PTH with carotid atherosclerosis or arterial stiffness in large community-based populations [16,20]. Moreover, to our knowledge, the associations between serum vitamin D or PTH and various subclinical atherosclerotic phenotypes, such as carotid IMT, plaques, luminal diameter, and PWV, have not been evaluated simultaneously. The aim of the current study was to investigate the independent association of serum vitamin D and PTH with multiple subclinical markers of atherosclerosis and suggest possible pathways underlying this association between these factors and CVD morbidity and mortality.

## Methods

### Study population

This study was conducted as a baseline survey during the Dong-gu Study in Korea [24]. A total of 9,260 subjects aged ≥50 years were enrolled in the baseline survey between April 2007 and July 2010. Of these, 848 subjects with coronary heart disease or stroke were excluded from the current study. After the exclusion of 54 subjects without serum vitamin D or PTH measurements, 141 individuals whose records lacked information regarding carotid ultrasonography or baPWV measurements, and 478 subjects with missing covariate information, 7,739 subjects (2,996 males and 4,743 females) were included in the current analysis. All participants provided informed consent for use of their data, and the study was conducted in accordance with the guidelines of the Declaration of Helsinki. The Institutional Review Board of Chonnam National University Hospital approved the study protocol (I-2008-05-056).

### Physical examination and interview

All participants underwent a standardized physical examination that was performed by well-trained research staff. Height, weight, and waist circumference were measured while the subjects were lightly dressed without shoes in the standing position. Body mass index (BMI) was calculated by dividing weight (kg) by height squared (m^2^). Blood pressure was measured using a customized cuff on the right upper arm with a mercury sphygmomanometer after 5-min rest in the sitting position. Three consecutive systolic and diastolic blood pressure readings were recorded at 1-min intervals, and the mean was used for the analysis. Information regarding demographics, smoking habits, alcohol consumption, medical history, and medications was collected from each subject by well-trained interviewers using a standardized questionnaire.

### Biochemical laboratory assessment

Blood samples were obtained from the antecubital vein in the morning after at least a 12-h overnight fast. Serum was separated on-site within 30 min using high-speed cold centrifuges, and the remaining samples were cryopreserved at −70°C until analysis. Lipid profiles and fasting glucose were measured using enzymatic techniques with an automated analyzer (Hitachi-7600, Hitachi Ltd., Tokyo, Japan). The estimated glomerular filtration rate (eGFR) was calculated as follows: 186.3 × (serum creatinine − 1.154) × (age − 0.203) × 0.742 (if female) [25]. The 25-hydroxyvitamin D (25-OHD), the primary stored form of vitamin D, and PTH concentrations were determined using chemiluminescent microparticle immunoassay (Architect *i*2000, Abbott, IL, USA). The coefficient of variation for the total analytical precision of this assay was ≤10% for 25-OHD and ≤9% for PTH. The lower detection limits of the assay were 3.0 ng/mL for 25-OHD and 1.0 pg/mL for PTH.

### Atherosclerotic parameters

The methods for carotid ultrasonography, image analysis, and confirmation of assay reproducibility were described previously [24]. Briefly, the carotid artery structure including, the IMT, plaques, and luminal diameter, was evaluated on the longitudinal view using a high-resolution B-mode ultrasound (Sonoace 9900, Medison, Seoul, Korea) equipped with a 7.5-MHz linear array transducer. Carotid IMT was defined as the mean of the maximum IMT of the left and right common carotid artery. Carotid plaques were defined as focal structures that encroached into the lumen by at least 100% of the surrounding IMT value. The carotid luminal diameter was determined as the mean of the luminal diameter of the left and right common carotid artery at a point 10 mm proximal to the carotid bulb origin.

The baPWV was determined using an automatic waveform analysis device (VP-1000, Colin Co., Komaki, Japan) after 5-min rest in supine position. Volume waveforms were obtained simultaneously from the brachial and tibial arteries for a sampling time of 10 s using automatic gain analysis and quality adjustment. The transmission time from the ascending point of the right brachial pulse volume recording to the ascending point of each ankle pulse volume recording was determined, and the transmission distance from the arm to each ankle was calculated according to body height. Then, the baPWV value was computed automatically as the transmission distance divided by the transmission time. The mean value of the left and right baPWV was used for the analyses.

### Statistical analyses

The characteristics of the study population were compared using Student’s *t*-tests for continuous variables, and χ^2^ tests for categorical variables. Vitamin D status was classified as severe deficiency (<10.0 ng/mL), deficiency (10.0–19.9 ng/mL), insufficiency (20.0–29.9 ng/mL), and sufficiency (≥30.0 ng/mL) according to serum 25-OHD concentrations. PTH concentrations were categorized into distribution-based quartiles. Multiple logistic regression analyses were used to evaluate the association between vitamin D and PTH concentration with carotid plaques. Adjusted odds ratio (OR) and 95% confidence intervals (CIs) for carotid plaques according to 25-OHD groups and PTH concentration quartiles were assessed. The mean (95%CI) value of carotid IMT, luminal diameter, and baPWV in the different 25-OHD groups and PTH quartiles were estimated using general linear models and compared using the group with the lowest serum vitamin D and PTH as a reference. The linear trends across the groups with increasing 25-OHD levels and increasing PTH quartiles were also assessed. Multiple logistic regression and general linear model analyses were performed sequentially in four models: model 1 was adjusted for gender and age; model 2 was model 1 with further adjustment for body mass index, calendar year, month of blood collection, current smoking, and current drinking; model 3 was model 2 with further adjustment for systolic blood pressure, fasting blood glucose, total cholesterol, high-density lipoprotein (HDL) cholesterol, eGFR, antihypertensive medication, anti-diabetic medication, and anti-dyslipidemic medication; and model 4 was model 3 with further adjustment for 25-OHD or PTH. All statistical analyses were performed using SPSS software version 22.0 (SPSS Inc., Chicago, IL, USA). *P*-value <0.05 was considered to indicate statistical significance.

## Results

### Baseline characteristics of the study population

The baseline characteristics of the study population according to 25-OHD and PTH are shown in Tables 1 and 2, respectively. The prevalences of vitamin D severe deficiency, insufficiency, and sufficiency were 7.8%, 67.7%, 21.6%, and 2.9%, respectively. BMI, WC, SBP, DBP, TC, TG, HDL-C, and phosphate levels showed an increasing trend as 25-OHD concentrations increased, whereas calcium showed a decreasing trend in this regard (*P*-trend < 0.05). The proportion of males, calcium supplement users, and multi-vitamin supplement users showed an increasing trend as 25-OHD concentrations increased, and the proportion of never-smokers, anti-diabetic medication users, and anti-dyslipidemic medication users showed a decreasing trend in this regard (*P*-trend < 0.05) (Table 1). Age, WC, SBP, DBP, calcium, and phosphate levels showed an increasing trend as PTH concentrations increased, whereas TC, TG, FBG, and eGFR levels showed a decreasing trend in this regard (*P*-trend <0.05). The proportion of never-smokers and anti-hypertensive medication users showed an increasing trend as PTH concentrations increased, and the proportion of males, current drinkers, anti-diabetic medication users, and multi-vitamin supplement users showed a decreasing trend in this regard (*P*-trend < 0.05) (Table 2).

**Table 1:** Baseline characteristics of study population according to serum 25-hydroxyvitamin D levels.

	Severe deficiency [<10.0 ng/mL] (n = 606)	Deficiency [10.0–19.9 ng/mL] (n = 5,237)	Insufficiency [20.0–29.9 ng/mL] (n = 1,674)	Sufficiency [≥30.0 ng/mL] (n = 222)	*P*-difference	*P*-trend
Male, n (%)	79 (13.0)	1,689 (32.3)	1,072 (64.0)	156 (70.3)	<0.01	0.11
Age, years	67 ± 9	65 ± 8	65 ± 8	66 ± 8	<0.01	0.11
Body mass index, kg/m^2^	24 ± 3	24 ± 3	24 ± 3	24 ± 3	<0.01	0.01
Waist circumference, cm	88 ± 10	88 ± 9	87 ± 8	86 ± 8	<0.01	<0.01
Systolic blood pressure, mmHg	125 ± 17	123 ± 17	122 ± 16	122 ± 16	<0.01	0.04
Diastolic blood pressure, mmHg	75 ± 11	74 ± 10	74 ± 10	74 ± 11	0.11	0.06
Total cholesterol, mg/dL	220 ± 44	204 ± 38	194 ± 37	187 ± 36	<0.01	<0.01
Triglycerides, mg/dL*	133 (93–188)	118 (85–172)	114 (79–167)	108 (75–162)	<0.01	<0.01
HDL cholesterol, mg/dL	54 ± 13	52 ± 12	51 ± 12	50 ± 13	<0.01	<0.01
Fasting blood glucose, mg/dL	108 ± 26	109 ± 25	109 ± 22	109 ± 22	0.76	0.85
eGFR, mL/min per 1.73m^2^	70 ± 14	70 ± 19	70 ± 12	70 ± 12	0.69	0.61
Calcium, mg/dL	7.7 ± 2.1	8.3 ± 1.8	8.4 ± 1.8	8.6 ± 1.6	<0.01	<0.01
Phosphate, mg/dL	3.9 ± 0.6	3.8 ± 0.6	3.6 ± 0.5	3.6 ± 0.5	<0.01	<0.01
Month of blood collection, n (%)					<0.01	<0.01
April	207 (34.2)	1,104 (21.1)	201 (12.0)	29 (13.1)		
May	239 (39.4)	1,683 (32.1)	447 (26.7)	42 (18.9)		
June	112 (18.5)	1,544 (29.5)	579 (34.6)	99 (44.6)		
July	48 (7.9)	906 (17.3)	447 (26.7)	52 (23.4)		
Smoking status, n (%)					<0.01	<0.01
Never	512 (84.5)	3,845 (73.4)	882 (52.7)	104 (46.8)		
Former	41 (6.8)	877 (16.7)	544 (32.5)	87 (39.2)		
Current	53 (8.7)	515 (9.8)	248 (14.8)	31 (14.0)		
Current drinking, n (%)	166 (27.4)	2,338 (44.6)	991 (59.2)	139 (62.6)	<0.01	<0.01
Anti-hypertensive medication use, n (%)	207 (34.2)	1,767 (33.7)	525 (31.4)	72 (32.4)	0.32	0.11
Anti-diabetic medication use, n (%)	82 (13.5)	621 (11.9)	171 (10.2)	20 (9.0)	0.07	<0.01
Anti-dyslipidemic medication use, n (%)	50 (8.3)	416 (7.9)	102 (6.1)	15 (6.8)	0.08	0.02
Calcium supplement use, n (%)	101 (16.7)	959 (18.3)	349 (20.8)	57 (25.7)	<0.01	<0.01
Multi-vitamin (including vitamin D) supplement use, n (%)	112 (18.5)	1,148 (21.9)	459 (27.4)	78 (35.1)	<0.01	<0.01

Data are means ± standard deviations or geometric means (interquartile range)* for continuous variables and frequencies (percentages) for categorical variables. HDL, high-density lipoprotein; eGFR, estimated glomerular filtration rate.

**Table 2:** Baseline characteristics of study population according to serum parathyroid hormone levels.

	First quartile [8.5–30.5 pg/mL] (n = 1,937)	Second quartile [30.6–39.3 pg/mL] (n = 1,939)	Third quartile [39.4–50.4 pg/mL] (n = 1,925)	Fourth quartile [50.5–340.4 pg/mL] (n = 1,938)	*P*-difference	*P*-trend
Male, n (%)	866 (44.7)	763 (39.4)	738 (38.3)	629 (32.5)	<0.01	<0.01
Age, years	64 ± 8	64 ± 8	65 ± 8	67 ± 8	<0.01	<0.01
Body mass index, kg/m^2^	24 ± 3	24 ± 3	24 ± 3	24 ± 3	0.15	0.06
Waist circumference, cm	88 ± 8	88 ± 9	88 ± 8	88 ± 9	<0.01	0.01
Systolic blood pressure, mmHg	122 ± 17	122 ± 16	124 ± 17	125 ± 17	<0.01	<0.01
Diastolic blood pressure, mmHg	73 ± 10	74 ± 10	75 ± 10	75 ± 11	<0.01	<0.01
Total cholesterol, mg/dL	204 ± 40	203 ± 38	201 ± 39	201 ± 39	0.17	0.04
Triglycerides, mg/dL*	124 (88–185)	117 (82–168)	116 (83–168)	115 (83–169)	<0.01	<0.01
HDL cholesterol, mg/dL	51 ± 12	52 ± 12	52 ± 13	52 ± 12	0.25	0.16
Fasting blood glucose, mg/dL	112 ± 28	109 ± 25	108 ± 23	107 ± 21	<0.01	<0.01
eGFR, mL/min per 1.73m^2^	72 ± 27	71 ± 12	70 ± 12	68 ± 14	<0.01	<0.01
Calcium, mg/dL	8.2 ± 2.0	8.3 ± 1.9	8.3 ± 1.8	8.4 ± 1.7	<0.01	<0.01
Phosphate, mg/dL	3.9 ± 0.6	3.8 ± 0.5	3.7 ± 0.5	3.7 ± 0.6	<0.01	<0.01
Month of blood collection, n (%)					<0.01	<0.01
April	392 (20.2)	380 (19.6)	378 (19.6)	391 (20.2)		
May	565 (29.2)	573 (29.6)	606 (31.5)	667 (34.4)		
June	560 (28.9)	602 (31.0)	579 (30.1)	593 (30.6)		
July	420 (21.7)	384 (19.8)	362 (18.8)	287 (14.8)		
Smoking status, n (%)					<0.01	<0.01
Never	1,255 (64.8)	1,353 (69.8)	1,350 (70.1)	1,385 (71.5)		
Former	434 (22.4)	386 (19.9)	377 (19.6)	352 (18.2)		
Current	248 (12.8)	200 (10.3)	198 (10.3)	201 (10.4)		
Current drinking, n (%)	991 (51.2)	956 (49.3)	915 (47.5)	772 (39.8)	<0.01	<0.01
Anti-hypertensive medication use, n (%)	575 (29.7)	585 (30.2)	654 (34.0)	757 (39.1)	<0.01	<0.01
Anti-diabetic medication use, n (%)	337 (17.4)	202 (10.4)	181 (9.4)	174 (9.0)	<0.01	<0.01
Anti-dyslipidemic medication use, n (%)	159 (8.2)	133 (6.9)	151 (7.8)	140 (7.2)	0.38	0.46
Calcium supplement use, n (%)	377 (19.5)	358 (18.5)	378 (19.6)	353 (18.2)	0.59	0.52
Multi-vitamin (including vitamin D) supplement use, n (%)	476 (24.6)	476 (24.5)	417 (21.7)	428 (22.1)	0.047	0.02

Data are means ± standard deviations or geometric means (interquartile range)* for continuous variables and frequencies (percentages) for categorical variables. HDL, high-density lipoprotein; eGFR, estimated glomerular filtration rate.

### Associations between 25-OHD and PTH with carotid plaques and carotid IMT

The associations between serum 25-OHD and PTH with carotid plaques according to multiple logistic regression analysis are shown in Table 3. There was no significant association between 25-OHD and carotid plaques. In addition, serum PTH was not significantly associated with carotid plaques in any of the models. The mean carotid IMT according to vitamin D status and PTH quartiles are presented in Table 4. No significant association was detected between serum vitamin D and carotid IMT (*P*-trend = 0.16 in model 4). Also, no significant linear trend was observed regarding the association between serum PTH and carotid IMT in any model (*P*-trend = 0.93 in model 4).

**Table 3:** Associations between serum 25-hydroxyvitamin D and parathyroid hormone levels with carotid plaque using multiple logistic regression analysis.

	Model 1	Model 2	Model 3	Model 4
	OR (95% CI)	OR (95% CI)	OR (95% CI)	OR (95% CI)
25-hydroxyvitamin D, ng/mL				
Severe deficiency [<10.0]	1.00	1.00	1.00	1.00
Deficiency [10.0–19.9]	0.92 (0.76–1.10)	0.91 (0.75–1.10)	0.96 (0.79–1.16)	0.98 (0.81–1.20)
Insufficiency [20.0–29.9]	0.90 (0.73–1.12)	0.91 (0.73–1.13)	1.01 (0.81–1.26)	1.07 (0.85–1.35)
Sufficiency [≥30.0]	1.01 (0.72–1.40)	1.01 (0.72–1.42)	1.14 (0.80–1.61)	1.23 (0.86–1.75)
Parathyroid hormone, pg/mL				
First quartile [8.5–30.5]	1.00	1.00	1.00	1.00
Second quartile [30.6–39.3]	0.98 (0.85–1.12)	0.96 (0.83–1.10)	0.98 (0.85–1.13)	1.01 (0.87–1.17)
Third quartile [39.4–50.4]	1.06 (0.93–1.22)	1.04 (0.90–1.19)	1.06 (0.92–1.22)	1.11 (0.96–1.29)
Fourth quartile [50.5–340.4]	1.06 (0.92–1.21)	1.00 (0.87–1.16)	1.02 (0.88–1.18)	1.09 (0.94–1.27)

OR, odds ratio; CI, confidence interval. Model 1 was adjusted for gender and age; model 2 was model 1 further adjusted for body mass index, calendar year, month of blood collection, smoking status, and current drinking; model 3 was model 2 further adjusted for systolic blood pressure, fasting blood glucose, total cholesterol, high-density lipoprotein cholesterol, estimated glomerular filtration rate, antihypertensive medication, anti-diabetic medication, and anti-dyslipidemic medication use; model 4 was model 3 further adjusted for calcium, phosphate, calcium supplement use, multi-vitamin (including vitamin D) use, and parathyroid hormone (for 25-hydroxyvitamin D), or 25-hydroxyvitamin D (for parathyroid hormone).

**Table 4:** Associations between serum 25-hydroxyvitamin D and parathyroid hormone with carotid IMT, carotid luminal diameter, and baPWV using general linear models.

	Model 1	Model 2	Model 3	Model 4
Carotid IMT, mm				
25-hydroxyvitamin D, ng/mL				
Severe deficiency [<10.0]	0.71 (0.70–0.73)	0.73 (0.72–0.74)	0.73 (0.72–0.75)	0.73 (0.72–0.74)
Deficiency [10.0–19.9]	0.72 (0.72–0.73)	0.74 (0.73–0.74)	0.74 (0.74–0.75)	0.74 (0.73–0.75)
Insufficiency [20.0–29.9]	0.73 (0.72–0.73)	0.74 (0.73–0.74)	0.75 (0.74–0.76)	0.75 (0.74–0.76)
Sufficiency [≥30.0]	0.72 (0.70–0.74)	0.73 (0.71–0.75)	0.75 (0.73–0.76)	0.74 (0.73–0.76)
*P-trend*	0.47	0.84	0.22	0.16
Parathyroid hormone, pg/mL				
First quartile [8.5–30.5]	0.73 (0.72–0.73)	0.74 (0.73–0.74)	0.74 (0.74–0.75)	0.74 (0.73–0.75)
Second quartile [30.6–39.3]	0.73 (0.72–0.73)	0.74 (0.73–0.74)	0.74 (0.74–0.75)	0.74 (0.73–0.75)
Third quartile [39.4–50.4]	0.73 (0.72–0.73)	0.74 (0.73–0.74)	0.75 (0.74–0.76)	0.74 (0.73–0.75)
Fourth quartile [50.5–340.4]	0.72 (0.71–0.72)*	0.73 (0.72–0.74)	0.74 (0.73–0.75)	0.74 (0.73–0.75)
*P-trend*	0.047	0.12	0.21	0.93
Carotid luminal diameter, mm				
25-hydroxyvitamin D				
Severe deficiency	6.27 (6.22–6.33)	6.32 (6.27–6.38)	6.35 (6.29–6.40)	6.32 (6.26–6.38)
Deficiency	6.28 (6.26–6.30)	6.33 (6.30–6.35)	6.34 (6.30–6.37)	6.33 (6.29–6.37)
Insufficiency	6.22 (6.19–6.25)	6.29 (6.26–6.33)	6.33 (6.28–6.37)	6.33 (6.28–6.37)
Sufficiency	6.21 (6.12–6.30)	6.32 (6.23–6.40)	6.33 (6.24–6.42)	6.34 (6.25–6.43)
*P-trend*	0.13	0.75	0.70	0.62
Parathyroid hormone				
First quartile	6.21 (6.18–6.24)	6.27 (6.24–6.30)	6.30 (6.26–6.34)	6.29 (6.25–6.33)
Second quartile	6.25 (6.21–6.28)	6.31 (6.28–6.34)*	6.34 (6.30–6.39)*	6.33 (6.28–6.37)*
Third quartile	6.27 (6.24–6.31)*	6.33 (6.30–6.36)*	6.35 (6.31–6.39)*	6.33 (6.29–6.38)*
Fourth quartile	6.32 (6.29–6.35)*	6.37 (6.34–6.40)*	6.37 (6.33–6.41)*	6.35 (6.31–6.40)*
*P-trend*	<0.01	<0.01	<0.01	<0.01
baPWV, cm/s				
25-hydroxyvitamin D				
Severe deficiency	1679 (1654–1704)	1654 (1628–1680)	1667 (1641–1692)	1669 (1642–1696)
Deficiency	1630 (1621–1639)	1631 (1620–1643)	1650 (1634–1665)	1656 (1639–1672)
Insufficiency	1586 (1571–1600)	1606 (1589–1622)	1645 (1626–1664)	1654 (1633–1674)
Sufficiency	1586 (1545–1626)*	1607 (1566–1648)*	1640 (1602–1677)	1650 (1612–1688)
*P-trend*	<0.01	0.03	0.19	0.38
Parathyroid hormone				
First quartile	1609 (1596–1623)	1613 (1598–1628)	1641 (1624–1659)	1644 (1626–1663)
Second quartile	1612 (1598–1626)	1618 (1602–1633)	1649 (1631–1667)	1653 (1634–1673)
Third quartile	1620 (1606–1634)	1627 (1611–1642)	1651 (1633–1670)	1656 (1637–1676)
Fourth quartile	1645 (1631–1659)*	1650 (1635–1665)*	1665 (1646–1683)*	1669 (1649–1689)*
*P-trend*	<0.01	<0.01	0.01	0.01

Data are means (95% confidence interval). IMT, carotid intima-media thickness; baPWV, brachial-ankle pulse wave velocity. Model 1 was adjusted for gender and age; model 2 was model 1 further adjusted for body mass index, calendar year, month of blood collection, smoking status, and current drinking; model 3 was model 2 further adjusted for systolic blood pressure, fasting blood glucose, total cholesterol, high-density lipoprotein cholesterol, estimated glomerular filtration rate, antihypertensive medication, anti-diabetic medication, and anti-dyslipidemic medication; model 4 was model 3 further adjusted for calcium, phosphate, calcium supplement use, multi-vitamin (including vitamin D) use, and parathyroid hormone (for 25-hydroxyvitamin D) or 25-hydroxyvitamin D (for parathyroid hormone).

*P< 0.05 compared to subjects with severe deficiency for 25-hydroxyvitamin D and subjects in the first quartile (8.5–30.5 pg/ml) for parathyroid hormone.

### Associations between 25-OHD and PTH with carotid luminal diameter and baPWV

Table 4 also shows the mean carotid luminal diameter and baPWV according to vitamin D status and PTH quartiles. Serum vitamin D was not significantly associated with carotid luminal diameter, whereas there was increasing trend between serum PTH and carotid luminal diameter in all models. The mean carotid luminal diameters were significantly greater in the second, third, and fourth PTH quartiles compared with the first quartile in the fully adjusted model (*P*-trend < 0.01 in model 4). Although the mean baPWV was significantly lower in individuals with vitamin D sufficiency compared with those with severe deficiency in models 1 and 2, these differences disappeared after further adjustment for covariates and serum PTH concentration in models 3 and 4. In contrast, mean baPWV increased with increasing PTH quartiles in all models. The mean baPWV was significantly greater in subjects with serum PTH levels in the fourth quartile compared with those in the first quartile in the fully adjusted model (*P*-trend = 0.01 in model 4).

## Discussion

In this large community-based cross-sectional study, we evaluated the association between serum 25-OHD and PTH concentration with subclinical atherosclerosis in middle-aged and elderly subjects aged ≥50 years. Serum PTH concentrations were positively associated with carotid luminal diameter and baPWV, but not with carotid IMT and plaques. However, there was no significant association between serum 25-OHD concentration and any atherosclerotic phenotypes, including carotid IMT, plaques, luminal diameter, and baPWV.

PTH secretion increases in response to low blood calcium levels because of vitamin D insufficiency [26]. In addition, PTH decreases quite linearly during vitamin D supplementation [27]. Although the underlying mechanisms behind these effects are not understood completely, epidemiological studies demonstrated that lower serum vitamin D and higher PTH levels were associated with cardiovascular morbidity and mortality significantly [1–6]. Recent prospective study identified interactions of vitamin D-related biomarkers with risk of coronary heart disease, showing that 25-OHD levels are inversely associated with coronary heart disease only among subjects with higher PTH levels [28]. To explain the link between vitamin D and PTH with CVD, recent studies examined the association between vitamin D, PTH, and subclinical atherosclerotic indices. Previous epidemiological studies investigated the relationship between vitamin D and carotid IMT, but the findings were inconsistent. Specifically, three studies found a lack of association between serum 25-OHD and carotid IMT [20–22]. Blondon et al. [20] observed no cross-sectional or longitudinal associations between serum 25-OHD and IMT of the common and internal carotid artery. However, a community-based study of 654 older adults reported a significant negative association between 25-OHD and internal carotid IMT, but no association with common carotid IMT [16]. The Northern Manhattan Study showed an inverse association between serum 25-OHD and carotid IMT among subjects with carotid plaques, although there was no association in the entire group [17]. Only a small number of studies have investigated the association between serum 25-OHD and carotid plaques, but all of these showed no significant association between serum 25-OHD with the presence, number, or incidence of plaques [17,20,21]. In addition, the role of vitamin D in reducing risk of CVD has not been found to be supported from recent experimental studies [29,30]. The published RCT results indicate that vitamin D supplements are ineffective in improving cardiovascular health among various patient populations [29]. In a Mendelian randomization design, no evidence to suggest that genetically reduced p-25(OH)D is associated with increased risk of ischemic heart disease was found [30]. In the current study, serum 25-OHD was not associated with carotid IMT or the presence of carotid plaques, consistent with the three previous studies [20–22].

Compared with previous reports analyzing 25-OHD, only a small number of studies have investigated the association of serum PTH with carotid IMT or plaques. Two population-based studies reported that serum PTH was not associated with carotid IMT and plaques [16,20], whereas another study reported that serum PTH was associated with carotid IMT in 107 postmenopausal females [23]. The current study showed no significant association between serum PTH and either carotid IMT or plaques. In this context, although limited data are available, neither serum 25-OHD nor PTH might be associated with carotid IMT or plaques. We were unable to explain the relationship between vitamin D, PTH, and CVD using structural vascular markers of carotid atherosclerosis such as IMT and plaques. Therefore, we could hypothesize that serum vitamin D and PTH might affect CVD via pathways not involving carotid atherosclerosis, such as IMT or plaques.

Previous studies demonstrated that arterial diameter was correlated with cardiovascular risk factors. Moreover, carotid enlargement was considered to be a surrogate marker of CVD [12,13]. The carotid artery might enlarge to preserve luminal area and stabilize the shear stress in response to arterial wall thickening and carotid plaque formation [31,32]. A larger lumen in diastole might reflect reduced intrinsic vessel elasticity and compliance, thereby suggesting a stiffer vessel. To our best knowledge, no epidemiological study has examined the association between serum vitamin D and PTH with carotid diameter. The current study revealed a positive association between serum PTH and carotid luminal diameter, but no association between serum 25-OHD and carotid luminal diameter. Therefore, serum PTH but not 25-OHD, might influence vascular compliance. Nevertheless, additional population-based studies are needed to confirm the associations between PTH and carotid luminal diameter.

In the present study, no association between serum 25-OHD and baPWV was observed. Previous studies examined the relationship between vitamin D and PWV, but the findings were inconsistent. Giallauria et al. [33] and Al Mheid et al. [19] showed that vitamin D insufficiency was associated with increased arterial stiffness using carotid-femoral PWV in healthy subjects, although the association was not adjusted for serum PTH concentrations. A significant inverse association between serum 25-OHD and PWV was also observed in 125 healthy subjects and 133 patients with newly diagnosed hypertension [34,35]. In contrast, recent studies reported no significant association between serum vitamin D and PWV [36,37]. Kruger et al. [36] did not observe an association between serum vitamin D and carotid-radial PWV in 291 urban females, and Pirro et al. [37] did not find an association between serum vitamin D and aortic PWV in 150 postmenopausal females. The results of the current study are consistent with those of several studies that reported no association between serum vitamin D and PWV [36,37].

To date, only two studies have examined the association between serum PTH and PWV, but the findings were contradictory. Pirro et al. demonstrated that serum PTH was significantly associated with aortic PWV, independent of established cardiovascular risk factors [37]. However, Giallauria et al. did not find a significant association between serum PTH and carotid-radial PWV [33]. In the current study, serum PTH levels were positively associated with baPWV, suggesting that serum PTH might decrease arterial compliance and increase arterial stiffness. In our study, PTH concentration increased with age, similar to a previous study [38]. PWV also increased with age. To eliminate confounding due to age, model 1 included age as an independent variable; the strength of the association between PTH and PWV was attenuated but remained statistically significant. This association also remained statistically significant after adjusting for 25-OHD and additional confounders. The discrepancies among studies might be explained in part by the different characteristics of each study group (age, gender, and number) and the different measurements used for arterial stiffness.

PWV and carotid luminal diameter are vascular compliance indices; therefore, an increased baPWV and an enlarged luminal diameter is associated with lower arterial compliance and stiffer vessels. This suggests that PTH might affect the development of atherosclerosis by altering vascular compliance. PTH secretion increases in response to low blood calcium levels because of vitamin D insufficiency. Thus, increased serum PTH concentrations due to vitamin D insufficiency might lead to low arterial compliance and high stiffness, resulting in increased CVD. High PTH was also associated with increased risk of heart failure and venous thromboembolism [39,40]. Clinical research has indicated that early treatment of parathyroid disorders may reverse cardiovascular remodeling and mitigate cardiac risk factors [41]. The pathophysiological mechanisms that link PTH to vascular compliance markers remain unclear. One plausible explanation is that PTH adversely affects vascular smooth muscle cells, which might increase vascular stiffness and promote atherosclerotic changes [42].

There are some limitations to the current study that must be considered. First, because of the cross-sectional design, we were unable to make causal inferences between serum 25-OHD, PTH and atherosclerotic markers. Second, serum 25-OHD and PTH were measured at a single time point, which does not reflect the long-term exposure. Long-term vitamin D deficiency should be considered an aspect of the CVD process [43,44] in consort with other factors, such as lipid levels, inflammation and subclinical infections/colonization, which may be influenced by vitamin D levels. Third, the effects of residual confounding variables such as vitamin D or calcium supplement intake cannot be excluded, although we adjusted for potential confounding variables. Fourth, recent studies have reported other pathways of vitamin D activation that generate alternative D3 hydroxyderivatives. No association between 25-OHD and atherosclerosis indices was found in this study, but additional assessment of the associations with alternative vitamin D3 hydroxyderivatives, such as 20-hydroxyvitamin D3 and atherosclerosis, should be considered [45–47]. Despite these limitations, this study has several strengths. First, it included a large community-based population compared with previous studies, which provided sufficient statistical power. Second, various subclinical atherosclerotic phenotypes that reflect different aspects of atherosclerosis, including carotid IMT, plaques, luminal diameter, and PWV, were evaluated simultaneously. Third, we evaluated not only the individual associations between serum 25-OHD and PTH with atherosclerotic phenotypes, but also the independent associations between serum 25-OHD and PTH with subclinical atherosclerosis.

## Conclusions

The current study revealed a positive association between PTH and vascular compliance parameters independent of conventional cardiovascular risk factors, but no association with structural vascular damage markers. There was no evidence of an association between serum 25-OHD and subclinical atherosclerotic phenotypes. These results suggest that PTH might affect the development of atherosclerosis, particularly by altering vascular compliance. Nevertheless, future studies are needed to confirm and further understand the association between PTH and vascular compliance markers.
